# Biocompatibility of Coronary Stents

**DOI:** 10.3390/ma7020769

**Published:** 2014-01-28

**Authors:** Thamarasee M. Jeewandara, Steven G. Wise, Martin K. C. Ng

**Affiliations:** 1 The Heart Research Institute, Sydney NSW 2042, Australia; E-Mails: thamarasee.jeewandara@hri.org.au (T.M.J.); wises@hri.org.au (S.G.W.); 2 Sydney Medical School, University of Sydney, Sydney NSW 2006, Australia; 3 School of Molecular Bioscience, University of Sydney, Sydney NSW 2006, Australia; 4 Department of Cardiology, Royal Prince Alfred Hospital, University of Sydney, Sydney NSW 2050, Australia

**Keywords:** coronary artery disease, biofunctionalization, stent, thrombosis, restenosis

## Abstract

Cardiovascular disease is the dominant cause of mortality in developed countries, with coronary artery disease (CAD) a predominant contributor. The development of stents to treat CAD was a significant innovation, facilitating effective percutaneous coronary revascularization. Coronary stents have evolved from bare metal compositions, to incorporate advances in pharmacological therapy in what are now known as drug eluting stents (DES). Deployment of a stent overcomes some limitations of balloon angioplasty alone, but provides an acute stimulus for thrombus formation and promotes neointimal hyperplasia. First generation DES effectively reduced in-stent restenosis, but profoundly delay healing and are susceptible to late stent thrombosis, leading to significant clinical complications in the long term. This review characterizes the development of coronary stents, detailing the incremental improvements, which aim to attenuate the major clinical complications of thrombosis and restenosis. Despite these enhancements, coronary stents remain fundamentally incompatible with the vasculature, an issue which has largely gone unaddressed. We highlight the latest modifications and research directions that promise to more holistically design coronary implants that are truly biocompatible.

## 1. Introduction

Cardiovascular disease continues to be the leading cause of mortality [1,2], with a vast majority of these deaths attributed to obstructive coronary artery disease (CAD) [3]. Depending on the severity of the disease, the main interventional options for revascularisation include angioplasty, stent deployment and in severe, diffuse occlusions (more than 70%), bypass graft surgery [3]. Narrowed coronary arteries were originally treated percutaneously with balloon angioplasty alone [4]. However, clinical complications including abrupt vessel closure from elastic recoil in the short term and significant neointimal hyperplasia, limited the applicability of this intervention. Improved results were observed following the insertion of an additional intravascular mechanical support, cylindrical metal scaffolds known as stents [5]. The first balloon expandable stents were designed from surgical grade stainless steel, and aimed to provide additional mechanical support, limiting vessel recoil and preventing acute occlusion [6]. Stents were initially evaluated in a preclinical study relative to angioplasty alone, in canine coronaries to assess efficacy prior to human trials [5,7]. The extent of endothelial damage during angioplasty is proportional to the time of balloon inflation [5]. Since the balloon is immediately deflated after maximal inflation during stent implantation and 80% of the expandable wire mesh of the stent was open surface opposing elastic recoil, the process minimized endothelial damage compared to balloon angioplasty alone [5]. The first human clinical implantations indicated a high delivery success, low incidence of perioperative complications and a thrombosis incidence controllable with the use of anticoagulants [8,9]. In the absence of antiplatelet therapy, sub-acute thrombotic closure after stent implantation, was a notable risk [8].

Despite some benefits over angioplasty alone, stent deployment still results in significant injury to the vessel wall and disruption of the endothelium [10]. Disruption of endothelial monolayer integrity induces a cascade of pro-inflammatory events resulting in monocytic infiltration and smooth muscle cell proliferation, which are key contributing factors to neointimal hyperplasia. The rate of re-endothelialization following injury is a critical determinant of vascular lesion formation and areas of injury that rapidly re-endothelialize have significantly less intimal thickening and restenosis [11], while also deterring thrombus formation [12]. In humans, bare metal stent struts are substantially endothelialized in 6–7 months, with significant coverage present after 2 months [13]. During this reformation of the endothelium over stent struts, the smooth muscle proliferation induced by injury contributes to neointimal formation and restenosis. The high rates of restenosis for bare metal stents are a significant drawback in their clinical application.

Preliminary drug-coated stents were engineered with surface anticoagulants, such as heparin or warfarin to prevent sub-acute thrombosis and bleeding complications [14]. Despite attenuating thrombosis, restenosis was unchanged, requiring a pharmacological approach for its inhibition. Drug eluting stents (DES) releasing anti-proliferative agents such as sirolimus and paclitaxel inhibit neointimal hyperplasia but also substantially delay healing and re-endothelialization [13]. Consequently, DES are not only susceptible to early thrombosis like bare metal stents (BMS), but are also prone to both late (30 days–1 year) and very late (>1 year) stent thrombosis [15]. In stable single vessel disease patients, late stent thrombosis (LST) occurs at a constant rate (0.6% per year) [16], with even higher rates reported (0.9%–3%/year) in real world studies [17]. Accordingly, the safety of DES remains in question [18]. Hence the advent of DES has further exacerbated the biocompatibility issues of coronary stent implantation. The unsatisfactory performance of both BMS and DES has led to continued investigation of novel stent modifications, focusing on improving stent biocompatibility. The innovations discussed include surface tissue engineering, endothelial regeneration mechanisms, nanotechnology, and plasma physics for the biofunctionalization of coronary stents.

## 2. Limitations of Bare Metal Stents (BMS)

The metal alloys used to produce bare metal stents are fundamentally incompatible with the vasculature, promoting thrombosis due to their inherent surface properties [19] while exerting no inhibitory effect on smooth muscle cell hyperproliferation. The dominant mode of early BMS failure is acute thrombosis, which can be as high as 24% in the absence of the dual anti-platelet therapy administered to stent recipients [8]. Stent thrombosis is defined as a composite 30-day endpoint, which can present as an abrupt vessel closure, large non-fatal myocardial infarction or death [20]. Deaths attributed to cardiac causes within the first 30 days of stent implantation are usually adjudicated as stent thrombosis [21]. Neointimal hyperplasia, or restenosis, is a major cause of bare metal stent failure after the early thrombosis risk has abated. In-stent restenosis is driven by an uncontrolled immune response, triggered by the disruption of the native endothelium and damage to the vessel wall. The re-modelling of the vessel post-injury is characterized by hyper-proliferative smooth muscle cells infiltrating into the vessel lumen and secreting extracellular matrix components [22].

BMS are made from surgical grade metal alloys, initially 316 L stainless steel (316 L SS), but more recently evolving to cobalt chromium and platinum alloys [23]. Stent strut thickness and alloy type play an integral role in the biological responses elicited. Changes to the metal alloy have facilitated thinner strut design while retaining sufficient radial strength, and led to the re-design of stent structures for increased deliverability. The first Palmaz-Schatz crown stent designed for flexibility has evolved significantly to the malleable S-shaped velocity-stent, currently in development [24]. Stent design has further developed to include the Multilink stents with still thinner struts, Microstents and GFX stents [25] made of sinusoidal element of stainless steel. Sub-acute thrombosis rates, post stent implantation, have greatly reduced over the course of stent evolution, although the rate of in-stent restenosis remained high [4].

## 3. Drug Eluting Stents (DES): An Imperfect Solution

Systemic drug administration post BMS implantation to reduce restenosis was ultimately unsuccessful due to low drug concentrations, non-specifically targeting the neointima [26]. DES locally releasing anti-proliferative agents were introduced in 2003 to reduce restenosis associated with stent implantation [27]. While DES have been highly effective in suppression of neointimal hyperplasia (up to 10-fold compared to BMS [28]) local vascular delivery of rampamycin analogues or paclitaxel is an untargeted approach, employing non-specific agents to inhibit all cell proliferation [29]. These drugs bind FK506 binding protein-12 (FKB12) which in turn blocks the cell-cycle specific kinase, mammalian target of rapamycin (mTOR), to halt mitotic progression in the juncture of G_1_ and S phases in all cell types [30,31]. This in turn deregulates tissue factor in endothelial cells and monocytes [32,33]. The elution of anti-proliferative agents is associated with a dramatic delay in healing and re-endothelialization at the stent deployment site; such that DES struts have less than 50% endothelial coverage at three years [13].

DES development has focused on the major failings of current devices and has included modifications to the metal alloys, coating polymers and eluted drugs [34]. For example, Abbott Vascular have developed a 2nd generation everolimus-eluting XIENCE V stent, using a different stent alloy (cobalt chromium), polymer coating (fluoropolymer) and anti-restenotic drug to its 1st generation counterparts. This resulted in enhanced endothelialization *in vitro* and *in vivo* compared to 1st generation DES [35]. In randomised clinical trials, the XIENCE V stent also exhibited improved safety outcomes compared to two iterations of first generation paclitaxel-eluting stents [17,36]. Other approaches for second generation DES include the use of biodegradable polymers and selective coating of the anti-restenotic drug solely on the abluminal surface of the stent [37]. Despite these innovations, significant rates of major adverse cardiac events persist, particularly in real world usage of DES incorporating a high proportion of patients with acute coronary syndromes [37].

The most recent innovations in DES development are combinations of existing technology; employing drug-elution from a resorbable stent platform [38], from an ultra-thin degradable polymer coating [39] or combined with endothelial cell capture [40]. These approaches are included in more detail in Section 5, below.

## 4. Underlying Causes of Stent Incompatibility

The compatibility of bare metal stents is due both to the stent material and design, while DES effectiveness is also affected by polymers used for coating and the anti-proliferative drugs released. Design considerations such as strut thickness, cell design and mechanical properties have been steadily optimized, while polymer coatings and drug effects remain problematic, increasing inflammation [41], delaying re-endothelialization [13] and impairing endothelial cell function [42].

### 4.1. Inherent Thrombogenicity

Stents are inherently foreign bodies in the vessel wall, inducing platelet adhesion and activating coagulation, leading to thrombosis. Inhibition of platelet activation is required following stent delivery. The currently low rates of early stent thrombosis (1%–2%) [43] are predicated on tolerance and adherence to dual antiplatelet therapy with aspirin and a thienopyridine. This is not feasible for an increasing number of patients with high bleeding risk, or those requiring surgery [44] and is associated with increased risk of significant morbidity including gastrointestinal bleeding [45]. There is also risk of antiplatelet hypo-responsiveness, which increases stent thrombosis [46]. In the case of DES, management of LST is additionally problematic. To reduce the incidence of late thrombotic events, extended dual anti-platelet therapy is now recommended following DES placement, though no consensus on the effectiveness of an extended regimen has been reached [47,48]. In a recent large cohort study, new generation DES (n-DES) provided a modest improvement in clinical outcomes compared to old generation DES (o-DES) [49]. Old DES classified in the study, were first generation DES with bare metal platforms and sirolimus or paclitaxel drug elution (Cypher, Cypher Select, Taxus Express, Taxus Liberté and Endeavor) [49]. New DES classified in the study diversely included; stents eluting non-inflammatory drug zotarolimus coupled with a biocompatible polymer system (bioLinx™) [50] designed to extend the duration of drug exposure in the vessel (Endeavor Resolute), multi-layer coating technology (Xience V) [51] and self-expanding stents designed for compression resistance (Promus Element) [52]. The study compared long-term outcomes of PCI with n-DES *vs*. o-DES and BMS to show comparatively lowered risk of restenosis, LST and mortality for n-DES, although no significant effect was observed for thrombosis [49]. The duration of recommended dual antiplatelet therapy to prevent thrombosis remains unchanged for both old and new generation DES in patients.

### 4.2. Delayed Re-Endothelialization

**Figure 1.**
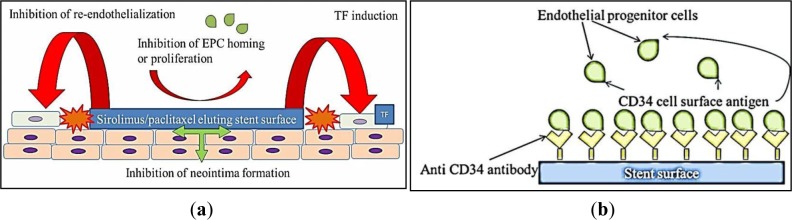

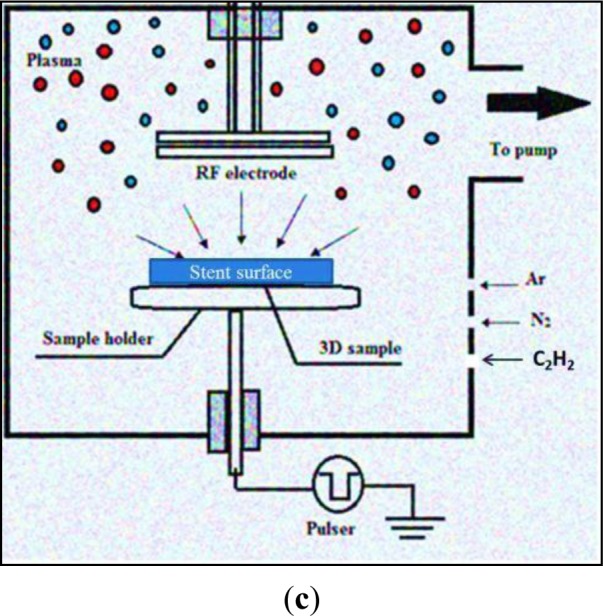
Coronary stent types, their mechanism of action and innovations: (**a**) first generation drug eluting stents (DES) impact on vascular biology: Modified from [42], reduced neointima formation (green arrows) but increased thrombogenicity at stent bio-interface. Sirolimus/paclitaxel inhibition of endothelialization, tissue factor induction and endothelial progenitor cells (EPC) homing prevention (red arrows); (**b**) EPC capture stent mechanism of action: Modified from [59]. The CD-34 antibody immobilized on the stent surface polymer binds the CD-34 antigen on the EPC to promote rapid endothelialization; (**c**) plasma surface modification for coronary stents: Modified from [60]. Nitrogen, argon and acetylene plasma is introduced into a chamber under a vacuum and ionized by a power source such as an RF electrode. The charged ions in the chamber impact the substrate to modify the surface immersed in the plasma.

As discussed above, speed of re-endothelialization is an important predictor of clinical outcome for stents. Following vascular injury, endothelial cells migrate from intact neighboring coronary segments, or are recruited from circulating endothelial progenitor cells (EPC) [53] to re-endothelialize the injured artery. However, both rapamycin and paclitaxel actively suppress endothelial cell growth *in vitro* [33,54,55] and impede EPC homing and proliferation *in vitro* [56,57], actively impeding re-endothelialization. A morphological autopsy study conducted to compare coronary artery segments from patients after DES and BMS implantation revealed delayed arterial healing and poorer endothelialization after DES compared to BMS implantation of similar duration [13]. Within the 1st generation DES cohort, 60% of patients had evidence of LST and a 45% rate of death was reported for patients suffering DES LST [13,58]. Re-endothelialization was significantly higher in BMS compared to DES [42]. The impacts of 1st generation DES on vascular biology are schematically represented in Figure 1a.

### 4.3. Metal and Polymer Coating Hypersensitivity

Hypersensitivity to metal alloys such as molybdenum and nickel has been previously observed in ~10% of patients undergoing BMS implantation [61] although the inflammatory response for stainless steel, is much less pronounced in comparison [62]. Hypersensitivity towards BMS alloys is associated with restenosis in the range of 15%–20% [42], with the extent of inflammation correlating to the degree of restenosis [63]. Marked hypersensitivity reactions have also been observed to the polymers coating DES. First generation DES coated with poly-ethylene vinyl acetate polymer are demonstrably pro-inflammatory [64]. This was further verified in a preclinical study when the copolymer, used as an antigen delivery matrix elicited an inflammatory response in ~25% of rabbits [65]. The inflammatory response in patients with spontaneous coronary dissection, post DES implantation was characterized primarily with eosinophilic infiltrations in the adventitia [63]. In severe cases DES related clinical complications exhibit necrotic core prolapse, in-stent restenosis and LST, preventing arterial healing [66]. A preclinical study in a porcine model showed progressive increases in the eosinophilic, granulomatous infiltrate, post first generation sirolimus (Cypher) stent implantation, starting at 28 days, increasing to 60% at 6 months [42].

### 4.4. Poor Coating Integrity

Another important, often overlooked aspect of stent safety is the coating integrity after crimping and expansion. Relatively few studies have evaluated the possibility of coating delamination [67,68], despite it being widespread in commercially available DES and recognized as a safety concern by the Food and Drug Administration [69]. DES polymer coatings display widespread surface cracking, peeling and flaking at the polymer-metal interface [70–72]; exposing the underlying thrombogenic metallic substrate and contributing to chronic inflammatory and hypersensitivity reactions [41,73].

Together, these failings highlight the difficulty in simultaneously promoting re-endothelialization, while inhibiting neointimal hyperplasia and thrombosis. No current stent platform adequately achieves this goal, but the latest strategies are reviewed below.

## 5. Novel Stent Modifications

Coatings aimed at increasing the inertness of metallic implants have been effective at reducing thrombogenicity but have generally failed to reduce restenosis rates. Examples of these coatings include gold [74]; diamond-like carbon [75]; pyrolytic carbon [76] and phosphorylcholine (PC). PC; exemplifying the flaws of an inertness approach was observed to be non-thrombogenic *in vitro* [77] however; *in vivo*; it failed to encourage endothelialization and ultimately had no effect on the rate of stent thrombosis [78]. In parallel; enhancement of stent biocompatibility has been pursued by actively influencing the host response. These coatings have failed because they only seek to address one aspect of vascular biocompatibility (e.g., thrombogenicity alone) at the expense of other aspects. Heparin-coated stents are one such example; designed to reduce thrombosis but not neointimal hyperplasia [79]. Below, we describe some of the most recent attempts to develop biocompatible stents.

### 5.1. Accelerating Endothelialization

Given that re-endothelialization plays an integral role in vascular healing after stent implantation, coating stents with substances to accelerate endothelial cell coverage is an important therapeutic approach [42]. Preliminary studies designed to capture endothelial progenitor cells (EPCs) by coating stents with a polysaccharide intermediate and murine monoclonal anti-human CD34-positive antibodies showed feasibility in human clinical trials [80]. The Genous Bio-engineered R stent, similarly coated with immobilized anti-CD34 antibodies aims to enhance endothelialization by capturing circulating endothelial progenitor cells (Figure 1b). The captured CD34-positive EPCs are proposed to differentiate into a mature endothelium, but the CD34-positive markers used are non-specifically shared by haematopoietic stem cells and immune complement cells. Circulating CD34-positive mononuclear cells are also shown to differentiate into smooth muscle progenitor cells in patients with restenosis [81]. A higher rate of revascularization was observed in Genous stent compared to Taxus stent, in patients treated for coronary artery stenosis with a high risk of restenosis [82]. A recent proof-of-concept study shows some benefits for endothelialization and thrombogenicity, but leaves reduction of neointimal hyperplasia unaddressed and the platform reliant on drug-eluting technology [83]. A novel DES coated with integrin-binding cyclic Arg-Gly-Asp peptides was similarly utilized in a preclinical study to accelerate endothelialization via EPC attraction, using the same principles of EPC capture. In an initial porcine model evaluation, neointimal hyperplasia seems to be promisingly reduced [84].

### 5.2. Bioresorbable Stents

Bioresorbable stents are proposed to solve the problem of long-term stent biocompatibility by degrading over time [85]. The first bioabsorbable, balloon expandable stents implanted in humans were constructed from poly-L-lactic acid (PLLA) [86]. The bonds between the repeating lactide units of the bioabsorbable stent hydrolyze to produce lactic acid, metabolized to CO_2_ and H_2_O [85]. Absorption occurs via bulk erosion throughout the implant not just on the surface, allowing the stent strut to retain its shape, until the process is well advanced [86]. The Abbott Vascular bioresorbable vascular scaffold (BVS), a PLLA stent, has so far demonstrated restenosis similar to bare metal platforms, as well as late scaffold shrinkage and non-uniform vessel support, due to uneven scaffold degradation [87]. Alloys of magnesium have also been explored as bioabsorbable stent platforms [88]. Absorption by surface erosion reduces the strut thickness as the stent is absorbed, within 4 months of implantation, leading to loss of radial support [88].

The latest generation bioabsorbable stents are designed for prolonged radial support coupled with drug elution [88]. A number of different materials have been utilized to manufacture these stents ranging from metal alloys to a variety of polymers, including tyrosine-derived polycarbonate polymer, salicylate and a linker, as well as metal-cobalt chromium with n-butyl methacrylate coating [89,90]. The BioMatrix stent incorporates the thin S-stent platform with a reduced percentage metal surface area (16.3%–18.4%) to elute the anti-proliferative drug biolimus A9 [39]; a highly lipophilic semi-synthetic analogue of sirolimus. Furthermore, the stent is completely bioabsorbable degrading *in vivo* to lactic acid in 6–9 months post implantation [38]. The JACTAX stent (Boston Scientific Corporation, Natick, MA, USA) was designed on similar principles, coated with an ultra-thin, mixture of biodegradable PLLA and paclitaxel drug applied as microdots, per 16-mm stent [91]. The stents were comparable to the preceding paclitaxel eluting stent (TAXUS Liberté, Boston Scientific Corporation, Natick, MA, USA), although further studies are underway to evaluate their potential for improved vessel healing. As yet, none have FDA approval for humans use, but some clinical trials are underway [92]. Early indications are that the technology remains problematic. Current bioresorbable stents have markedly inferior mechanical properties in terms of device profile, flexibility, deliverability and radial strength, thereby dramatically limiting their capacity to be used for a large number of coronary lesion subsets including bifurcation lesions, calcified lesions, tortuous coronaries and long lesions [93]. As a consequence of these profound limitations, metal alloy stents will remain the mainstay for endovascular stents in the foreseeable future.

### 5.3. Nanotechnology for Controlled Release of Drugs and Novel Stent Design for Myocardial Reperfusion

Novel mechanisms of drug release include polymeric nanoparticles (NPs) to encapsulate pharmaceutical agents for targeted drug delivery to a tissue of interest [94–96]. For instance, d-α-tocopheryl polyethylene glycol 1000 succinate (TPGS) is used as an emulsifier in the formulation of the biomaterial matrix poly(DL-lactide-co-glycolide) (PLGA). The biodegradable PLGA/TPGS NPs deliver controlled paclitaxel release with high drug encapsulation efficiency (EE), for the treatment of restenosis. The higher drug EE improves cellular uptake and cytotoxicity against SMC proliferation, and is being considered in the development of third generation, nanoparticle coated DES [97]. The Nevo-sirolimus eluting stent is designed from L605 cobalt-chromium alloy with a drug delivery system based on PLGA. It utilizes a multi-channel reservoir system along the stent struts into which the drug-polymer (sirolimus/PLGA) matrix is loaded for elution [98] displaying superiority over traditional paclitaxel eluting stents (TAXUS Liberté) in clinical trials [99].

In patients with acute ST-segment elevation myocardial infarction (STEMI) undergoing PCI, sub-optimal myocardial reperfusion is common. Stents have therefore been specifically designed to prevent thrombus protrusion into the lumen after PCI, in acute myocardial infarction. The potential utility of a novel polyethylene terephthalate (PET) micronet mesh-covered thin-strut metal stent (MGaurd), was evaluated in this regard for its functional design to trap and exclude thrombus and atheromatous debris to prevent distal embolization [100]. The stent showed superior rates of epicardial coronary flow and complete ST-segment resolution, compared to conventional metal stents. Larger clinical studies are required to determine improved clinical outcomes.

### 5.4. Plasma Polymerization

Plasma, the fourth state of matter is artificially generated when a dielectric gas ionized by free electrons is accelerated in a sufficiently strong electric field. The gas molecules separate on ion-impact, to create electrons and neutral gas atoms within a vacuum evacuated chamber. Subsequent collisions among these particles create more ions and electrons to interact with and modify the surfaces, including potential biomaterials [101,102], modifying their surface energy, charge and surface chemistry, without altering bulk properties [60]. When argon and an increasing amount of acetylene are used in a plasma deposition system, a plasma activated coating (PAC) was created on a metallic biomaterial surface (Figure 1c) suitable for stent applications. The thin carbon polymer layer on the biomaterial acts as a reservoir of free radicals, activating the surface for effective, covalent protein attachment [103,104].

Pulse biased plasma polymerization has been adapted to metallic substrates, for one step covalent biomolecule immobilization, minimising the otherwise complex process of chemical linker based biofunctionalization discussed elsewhere [105]. The relationship between protein binding and biomolecule activity on PAC strongly adhered to stainless steel, has been demonstrated by direct covalent attachment of tropoelastin, horseradish peroxidase and catalase [103,106]. Surface characterization further elucidated the importance of surface free energy for effective biomolecule attachment [60]. Recent evidence suggests that PAC on a 316 L SS stent covalently binds a dense layer of human recombinant tropoelastin, facilitating the growth of endothelial cells [107]. Plasma modified surfaces coated with tropoelastin have shown improved blood biocompatibility, significantly reduced clot formation and improved endothelialization *in vitro* [107]. Pre-clinical studies of PAC stents demonstrate feasibility and delivery with great potential as a carrier for local biomolecule delivery [108].

## 6. Conclusions and Future Perspectives

The development of coronary stents is an evolving process and a fundamental aspect of interventional therapy in the treatment of coronary artery disease. Stent biocompatibility is a multi-faceted process; having to be simultaneously hemocompatible, promote rapid re-endothelialization and suppress restenosis. BMS have evolved considerably since their first human use, but remain both thrombogenic and susceptible to restenosis. DES elute powerful cytotoxic drugs to inhibit SMC hyper-proliferation, but delay healing and induce inflammation, resulting in an increased risk of late thrombosis. We have highlighted the latest strategies that appear to be most promising, including active promotion of re-endothelialization, bioresorbable stents, nanotechnology and plasma based modification. In light of the limitations still evident for each of these approaches, further development is required, with biofunctionalized combination devices (e.g., bioresorbable drug-eluting stents) and local biomolecule delivery the most likely to have success. Overall, the many stent design innovations currently in development promise to address the underlying lack of biointegration more directly, on the path to a truly biocompatible stent.
